# Diagnostic Performance of Three-Phase Bone Scintigraphy and Digital Infrared Thermography Imaging for Chronic Post-Traumatic Complex Regional Pain Syndrome

**DOI:** 10.3390/diagnostics11081459

**Published:** 2021-08-12

**Authors:** Miju Cheon, Hyo Jung Kang, Kyung Hee Do, Hee Seung Yang, Eul Joo Han, Jang Yoo

**Affiliations:** 1 Veterans Health Service Medical Center, Department of Nuclear Medicine, Seoul 05368, Korea; jang8214.yoo@gmail.com; 2 Veterans Health Service Medical Center, Department of Physical Medicine and Rehabilitation, Seoul 05368, Korea; rehab@bohun.or.kr (H.J.K.); khdo@bohun.or.kr (K.H.D.); yang7310@bohun.or.kr (H.S.Y.); 3 Veterans Health Service Medical Center, Department of Oriental Medicine, Seoul 05368, Korea; woojicool@bohun.or.kr

**Keywords:** pain, complex regional pain syndrome, three-phase bone scintigraphy, digital infrared thermography

## Abstract

This study aimed to evaluate the diagnostic performance of three-phase bone scintigraphy (TPBS) and digital infrared thermography imaging (DITI) in the chronic post-traumatic CRPS and propose new imaging diagnostic criteria that combine the two tests. We retrospectively enrolled 44 patients with suspected symptoms of CRPS from various injuries during obligatory military service. We analyzed the following findings: (1) uptake pattern on TPBS, (2) uptake ratios of affected and unaffected sides in each phase of TPBS, (3) difference in body skin temperature on DITI. New criteria combining the above findings were also evaluated. Eighteen patients were finally defined as CRPS according to the Budapest criteria. Uptake pattern and uptake ratio in blood pool phase on the TPBS were significantly different between CRPS and non-CRPS groups (both *p* < 0.05). The DITI could not discriminate significantly between the groups (*p* = 0.334). The diagnostic criteria considering both the pattern analysis and quantitative analysis in TPBS exhibited the highest positive likelihood ratio. On the other hand, the diagnostic criteria combining DITI and TPBS showed the lowest negative likelihood ratio value. TPBS can be useful in diagnosing chronic post-traumatic CRPS. Moreover, we can suggest that different diagnostic criteria be applied depending on the purpose.

## 1. Introduction

Complex regional pain syndrome (CPRS) is clinically a disease in which symptoms such as diffuse pain, hypersensitivity, edema, and decreased autonomic and motor functions are present in the affected limb [1]. It can be caused by trauma, fractures, nerve damage of limbs, brain lesions including strokes, brain tumors, traumatic brain injury, spinal cord injury, myocardial infarction, and unspecified causes [2]. CRPS is one of the most misunderstood, over-diagnosed, under-diagnosed, and undiagnosed diseases in medicine. The diagnosis is based on clinical manifestations because the pathophysiology appears to be highly complex and difficult to elucidate [3,4]. Disease progression is variable over time, and not all patients progressed from an initial pain phase to an edema phase followed by a trophic phase [3]. Sometimes progression involves motor symptoms, including weakness, bradykinesia, dystonia, myoclonus, and tremor that could occur in chronic phases [1,5,6]. In clinical practice, the diagnosis of chronic CRPS is frequently difficult because there are no unique and characteristic features that distinguish CRPS from similar conditions [4,7]. Although bisphosphonate treatment is effective in the early stage, there is no proven effective treatment in the chronic stage. Nevertheless, some medications, physical therapy, occupational therapy, procedures such as sympathetic nerve blocks or neuromodulation can control the pain and improve the patient’s quality of life, so accurate CRPS diagnosis in the chronic stage is important [3,4,8]. 

As a method for diagnosing CRPS, simple x-rays, three-phase bone scintigraphy (TPBS), digital infrared thermography imaging (DITI), measurement of sweating, diagnostic sympathetic nerve block, and electromyography can be used [4]. However, no absolute diagnostic standard for CRPS in the form of an objective test has been established. Three-phase bone scintigraphy (TPBS) has been used in the clinical diagnostic evaluation of CRPS since the 1980s, although its value is controversial. One meta-analysis showed that the mean sensitivity of TPBS in twelve studies was 0.87 (95% CI, 0.68–0.97) and specificity was 0.69 (95% CI, 0.47–0.85) [7]. Another meta-analysis found that disease duration could make considerable changes to both the accuracy and threshold [9]. Veldman and colleagues ascertained that people with longstanding CRPS were more likely to have a cold limb. However, several early stage patients also presented with cold limbs, while many chronic stage patients had warm limbs [10]. In the chronic stage of the disease, changes in TPBS can exhibit a diverse pattern of radioactive uptake, such as increased, decreased, or normal radioactivity, throughout all phases [11,12]. This is similar to the variability of symptoms seen in the study by Veldman and colleagues. Therefore, the diagnostic ability of TPBS decreases in the chronic stage of the disease. In previous studies on CRPS, the sensitivity of DITI was reported to be from 32% to 94% and specificity was from 80% to 100% [13,14]. In a study on symptom duration and skin temperature asymmetry, there was no significant correlation between the duration of symptoms and differences in skin temperature between affected and unaffected limbs [15].

A multidisciplinary approach including diagnostic criteria that combines several objective tests is highly recommended, especially in the chronic stages of the disease, because of its often-debilitating course and refractory nature to treatment [3]. Very few studies that compare the usefulness of TBPS and DITI, and only two studies were conducted in Korea [15,16]. However, the number of target patients was not significant. In addition, most of the previous studies analyzed various causes of CRPS (stroke, trauma, head injury) and the duration of acute, subacute, and chronic disease without distinguishing between them [15,16,17,18]. There have been no studies that targeted only trauma or only chronic patients with duration of more than one year. Therefore, it is necessary to conduct a study with a more significant number of patients and targeted only chronic post-traumatic CRPS.

The current study aimed to evaluate the diagnostic performance of TPBS and DITI in the chronic post-traumatic CRPS and propose new imaging diagnostic criteria that combine the two tests. 

## 2. Materials and Methods

### 2.1. Subjects

We retrospectively reviewed medical records of 52 patients who visited the physical examination center for veterans’ qualifications in our hospital with at least one symptom in all four symptom categories (sensory, vasomotor, sudomotor/edema, and motor/trophic) of Budapest criteria [19,20] from April 2017 to September 2019. Physical examination was only possible if it is caused by various injuries that occurred during obligatory military service. Among them, patients who did not undergo both TPBS and DITI, patients with less than one year after symptom onset, and patients with bilateral pain in their extremities were excluded from the study. The Human Investigation Committee (IRB) of Veterans Health Service Medical Center approved this study (IRB no. 2020-02-022), approved on 25 February 2020. Informed consent from participants included in the study was obtained to publish the images. The final clinical diagnosis results were classified into CRPS and non-CRPS groups according to the Budapest criteria. The assessment was performed by physical medicine and rehabilitation physicians (HJK, KHD, HSY) blinded to the results of the TPBS and DITI.

### 2.2. Three-Phase Bone Scintigraphy

The TPBS was performed using the NM 630 SPECT gamma camera (GE Healthcare, Milwaukee, WI, USA) equipped with a low energy high resolution (LEHR) collimator (acquisition matrix 64 × 64 pixels for the blood flow phase and 128 × 128 pixels for the blood pool and delayed phase; energy window centered at 140 KeV; width 15%). After an intravenous bolus injection of 750 MBq Tc-99m MDP (methylene diphosphonate) into the forearm or leg vein on the unaffected side, a sequence of bilateral images was recorded for 0 to 180 s per every 2 s as the blood flow phase (phase 1). Immediately after the image, the position of the relevant part, such as the hand, was not changed, and the blood pool image was taken for 3 min (phase 2), and the delayed image was obtained after 4 h (phase 3). 

**Figure 1:**
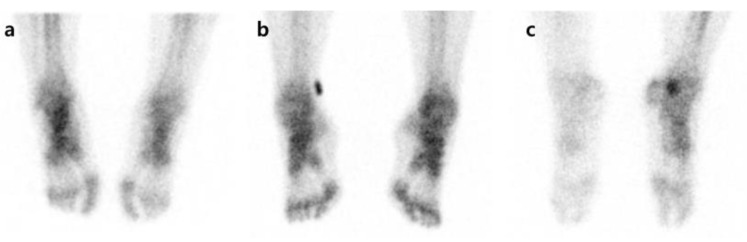
Examples of uptake pattern in three-phase bone scintigraphy. All patients have symptoms in the right foot. (**a**) Increased uptake on affected side, (**b**) no difference between affected and unaffected sides, (**c**) decreased uptake on affected side.

Pattern analysis was carried out by comparing degrees of radiotracer uptake in the affected side with the unaffected side for each phase. First, in each phase, an increase in radioactivity in affected extremities compared with contralateral extremities was defined as I. When the unaffected side and the affected side showed symmetric uptake, it was defined as S, and when the uptake in the affected side decreased compared to the unaffected side, it was defined as D (Figure 1). Overall, 27 patterns (I-I-I, I-I-S, I-I-D, I-S-I, I-S-S, I-S-D, I-D-I, I-D-S, I-D-D, S-I-I, S-I-D, S-I-S, S-D-I, S-D-S, S-D-D, S-S-S, S-S-D, S-S-I, D-I-I, D-I-S, D-I-D, D-S-I, D-S-S, D-S-D, D-D-I, D-D-S, and D-D-D) were created by combining the uptake patterns of each phase. An increase or decrease in the radiotracer uptake of the affected side compared to the unaffected side in phases 1, 2, or 3 was defined as an abnormal finding. As there is no typical pattern to diagnose CRPS by TPBS, we followed the method by Moon et al. [21]. After the pattern analysis, the cases showing I-I-I, S-S-I, D-D-I, D-D-S, and D-D-D patterns were determined as the CRPS group. If the radioactive uptake pattern was locally increased rather than in a diffuse way, it was regarded as a change due to a disease unrelated to CRPS, such as arthritis, and was considered as non-CRPS [22]. 

For quantitative analysis, regions of interest (ROIs) of the same size were set manually in each phase on the affected and unaffected side of the injured site. For example, in the case of the hand, it was set to include the carpal, metacarpal, and lumbar finger joints except for the fingers. In each phase, the total count in the ROI on the affected and unaffected sides was measured, and the ratio between the affected and unaffected sides was calculated. The TPBS images were reviewed and analyzed by two nuclear medicine physicians (MC and JY with 17 and 16 years of experience) who were utterly unaware of the clinical diagnosis.

### 2.3. Digital Infrared Thermography Images

**Figure 2:**
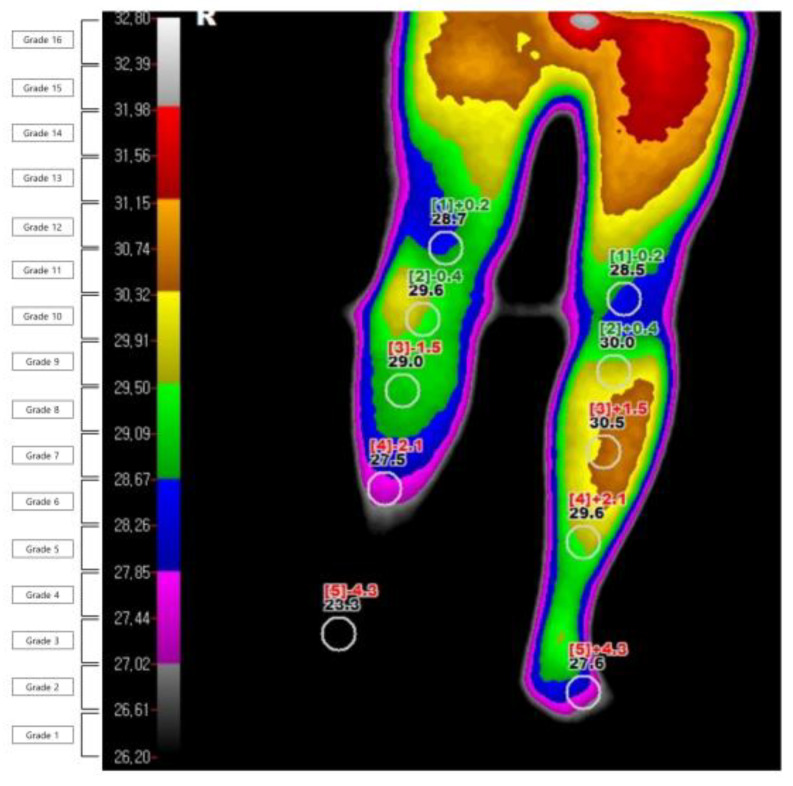
Example of digital infrared thermography imaging in a patient with complex regional pain syndrome.

The DITI were performed using an infrared thermography system (T-1000SMART, MESH Co., Ltd., Gangwon-do, Korea). The investigation was carried out in a temperature-controlled room at 19–21 °C. It was conducted after the subject was acclimatized to the room temperature for 15 min without contact and with their shirt removed. From the day before the test, all tests and treatments that can irritate the skin were avoided, and drugs that could affect the sympathetic nervous system were stopped. The degree of infrared emission was expressed in pixels through a computer monitor, and the body surface temperature was expressed visually through a total of 16 color grades (grade 1 to 16). The grade difference between the affected and unaffected sides was calculated (Figure 2). The diagnosis of CRPS through DITI was defined as a difference in one or more color grades in the ROI on the affected and unaffected sides of the image taken from the patients’ front. This threshold was based on former studies [10,23]. They were defined and read by one oriental medicine physician (EJH) and one physical medicine.

### 2.4. Combined Criteria

To establish the imaging diagnostic criteria that can improve the diagnostic performance for diagnosing chronic post-traumatic CRPS, two combined criteria were set, and the differences in diagnostic performance were compared and analyzed. We analyzed the following combinations: (1) It was considered CRPS even if there was an uptake pattern of I-I-I, S-S-I, D-D-I, D-D-S, or D-D-D in TPBS or a difference in one or more color grades in DITI, (2) It was considered as CRPS when an uptake pattern of I-I-I, S-S-I, D-D-I, D-D-S, or D-D-D was obtained and ratio on blood pool phase ≤ 0.805 in TPBS. Our primary outcome is to evaluate the diagnostic accuracy of TPBS and DITI in the chronic post-traumatic CRPS. The second outcome is to assess the diagnostic accuracy of new imaging diagnostic criteria that combine two tests.

### 2.5. Statistical Analysis

Statistical analyses were performed using SPSS version 18.0 software for Windows (SPSS Inc., Chicago, IL, USA). Descriptive statistics were reported as means and standard deviations, and continuous variables were reported as medians and standard deviations. Categorical variables were reported as frequencies and percentages. Comparisons of values between groups were performed using independent samples t-test for continuous variables and the Fisher’s exact test or Mann–Whitney U-test for dichotomous variables. Differences in diagnostic efficacy of each significant finding for differentiation between CRPS and non-CRPS were compared using McNemar’s test. Receiver operating characteristics (ROC) curve analysis was performed to measure the accuracy of cut-off values of significant criteria used to differentiate CRPS from non-CRPS. The diagnostic accuracy was expressed as the area under the corresponding ROC curve (AUC). The corresponding sensitivity (SN), specificity (SP), accuracy (AC), positive likelihood ratio (PLR), and negative likelihood ratio (NLR) were calculated for each finding. A *p* value of less than 0.05 was considered to indicate a statistically significant difference for all analyses.

## 3. Results

**Figure 3:**
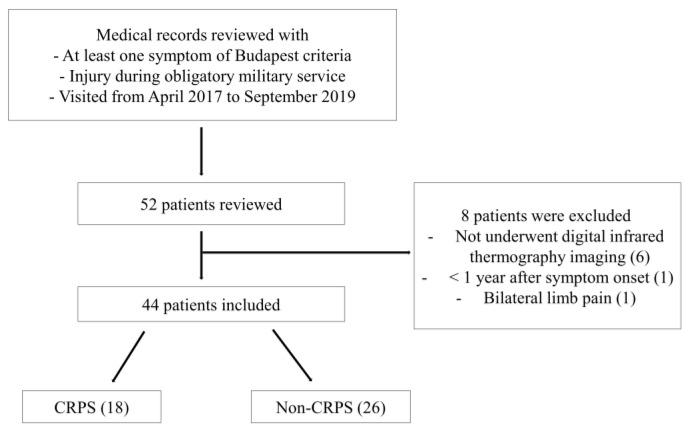
Flow diagram showing the inclusion process of patients in the study. CRPS, complex regional pain syndrome.

Of 52 patients, six patients who did not undergo infrared thermography, one patient less than one year after symptom onset, and one patient with bilateral pain in their limbs were excluded, and 44 patients were finally analyzed (Figure 3). The 44 patients were all male, and their mean age was 27.2 ± 4.1 years (21–37 years) (Table 1). Of the 44 patients, 18 patients (40.9%, 18/44) were finally defined as CRPS according to the Budapest criteria. As a result of our medical record review, no patients received bisphosphonate therapy or invasive therapies like sympathetic blockade before the examination. Some patients only performed nonsteroidal anti-inflammatory drugs, physical therapy, or ergotherapy.

The period from an injury during obligatory military service to the TPBS was 1149.7 ± 729.9 days. The average interval between the date of infrared thermography and the TPBS was 15.9 ± 26.1 days. The most common sites associated with injuries and symptoms were the foot and ankle (*n* = 26), the lower leg in 6, the hand in 5, the knee in 3, and the elbow in 1.

**Figure 4:**
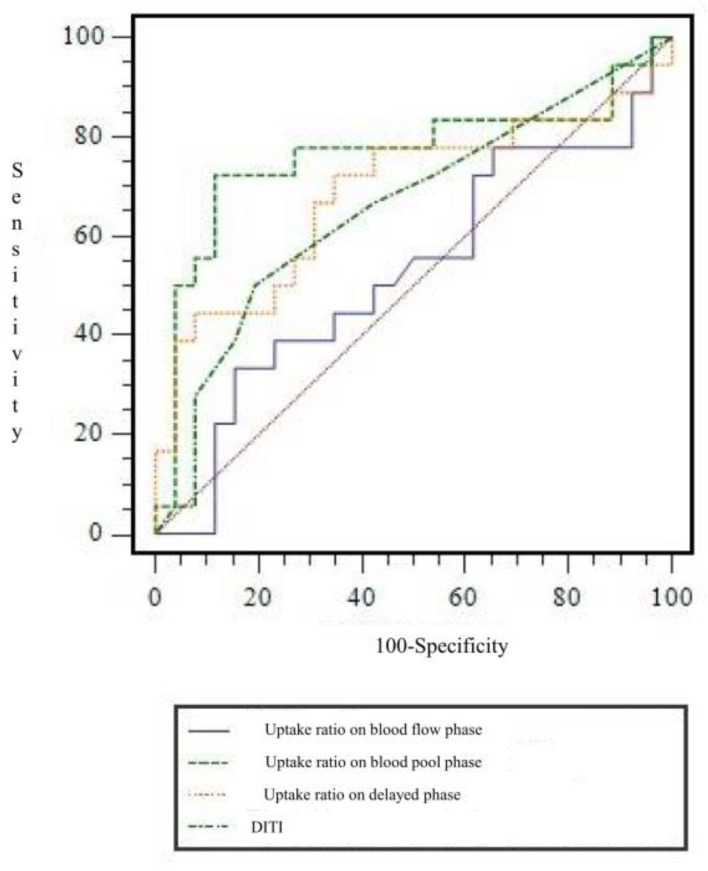
Receiver operating characteristic curve of uptake ratio on each phases in three-phase bone scintigraphy and digital infrared thermography imaging for diagnosing chronic post-traumatic complex regional pain syndrome. AUC for uptake ratio on blood flow phase, blood pool phase and delayed phase, and digital infrared thermography imaging were 0.527 ± 0.093, 0.763 ± 0.086, 0.686 ± 0.090, and 0.658 ± 0.085, respectively. AUC = area under curve.

In TPBS, when I-I-I, S-S-I, D-D-I, D-D-S, and D-D-D were used to determine CRPS among the combinations of uptake patterns, the CRPS and non-CRPS groups could be distinguished statistically significantly (*p* = 0.0016, Table 2). In the CRPS group, uptake patterns were the most common in the following order: D-D-D (*n* = 10), S-S-S (*n* = 3), S-S-I (*n* = 2), I-I-I (*n* = 2), and D-D-S (*n* = 1). As a result of quantitative analysis using count ratio, the uptake ratio in the blood pool phase showed a significant difference between the CRPS and non-CRPS groups (*p* = 0.011). In other words, in TPBS of patients with suspected CRPS at a time point more than one year after injury, the uptake in the blood pool of the affected side compared to the unaffected side was significantly reduced compared to that of the non-CRPS group. However, there was no significant difference between the two groups in terms of uptake ratio in blood flow (phase 1) and delayed phases (phase 3) (Table 3). ROC curve analysis was performed to find the cut-off value for the uptake ratio in the blood pool image, which was identified as a significant quantitative factor in diagnosing CRPS (Figure 4). The AUC value was 0.763. When a cut-off value for uptake ratio on blood flow phase was set at ≤0.805, the SN, SP, AC, PLR, and NLR for diagnosing CRPS were 72.2% (13/18), 88.5% (23/26), 81.8% (36/44), 6.259 (95% CI, 2.079 to 18.845), and 0.314 (95% CI, 0.147 to 0.670), respectively. 

In DITI, when the diagnosis of CRPS was defined as a difference in one or more color grades in the ROI on the affected and unaffected sides, the DITI could not discriminate significantly between the CRPS and non-CRPS groups (*p* = 0.334) (Table 2). In the patient group, when compared with the final results, there was one case that showed abnormal findings only on the DITI, and 6 cases that showed abnormal findings only with TPBS. 

**Figure 5:**
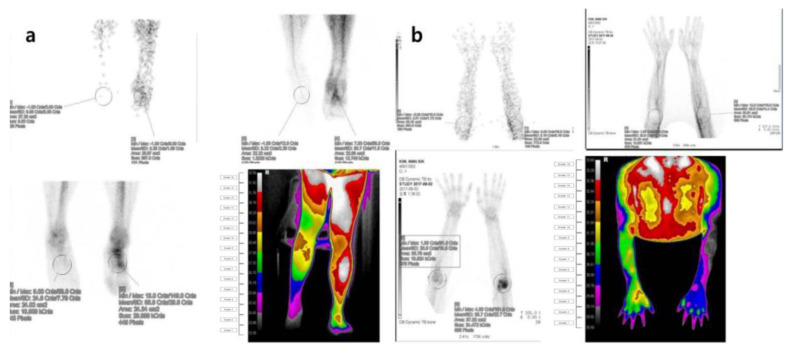
Three-phase bone scintigraphy and digital infrared thermography imaging in patients with suspected symptoms of complex regional pain syndrome (**a**) Three-phase bone scan and digital infrared thermography findings in a 21-year-old male complaining of persistent pain and skin changes after a right ankle sprain that occurred during training two years ago. The uptake of the right ankle was decreased in the blood flow, blood pool, and delayed image of the three-phase bone scan. The body surface temperature of the right ankle was decreased compared to the unaffected area in the digital infrared thermography. (**b**) Three-phase bone scan and digital infrared thermography findings in a 24-year-old male with a history of direct trauma to the left elbow three years ago. In a three-phase bone scan, blood flow, blood pool, and delayed intake in the left elbow area were increased, resulting in a positive finding. In infrared thermography, the body surface temperature at both elbows showed a positive finding with the difference in one more color grade.

In TPBS, both pattern analysis and quantitative analysis using count ratio showed valuable results, but the quantitative analysis showed significantly higher specificity (*p* = 0.0106) than pattern analysis. Although DITI did not show significant results in diagnosing chronic post-traumatic CRPS, there was a case with abnormal findings only in DITI, so we further analyzed with the diagnostic criteria combining DITI and TPBS. Further analysis was done to determine whether a combination of the significant diagnostic criteria could improve the efficacy of diagnosing CRPS. The diagnostic criteria with the highest PLR (7.94, 95%CI 1.995 to 31.629, *p* = 0.0002) were the combined 2 criteria (uptake pattern of I-I-I, S-S-I, D-D-I, D-D-S, or D-D-D and ratio on blood pool phase ≤0.805 in TPBS), which had significantly higher SP than qualitative analysis of TPBS (*p* = 0.0021). Additionally, it had significantly higher SP (*p* < 0.0001) and AC (*p* = 0.0386) and significantly lower sensitivity (*p* = 0.0002) than combined criteria 1 (uptake pattern of I-I-I, S-S-I, D-D-I, D-D-S, or D-D-D in TPBS or a difference in one or more color grades in DITI). However, there was no significant difference in SN (*p* = 0.2717), SP (*p* = 0.5429), and AC (*p* = 0.7883) between the criteria using quantitative analysis of TPBS and combined criteria 2. On the other hand, the diagnostic criteria combining DITI and TPBS (combined criteria 1) showed the lowest NLR value (0.160, 95%CI 0.222 to 1.159, *p* = 0.0020). The diagnostic performance of each finding for diagnosing CRPS is presented in Table 4. A representative case is presented as a figure (Figure 5).

## 4. Discussion

CRPS is a debilitating, painful condition in a limb associated with sensory, motor, autonomic, skin, and bone abnormalities [10]. CRPS commonly arises after injury to that limb. However, there is no relationship to the severity of the trauma, and in some cases, there is no precipitating trauma at all. Our findings were also consistent with this. The most common cause of persistent pain in our study was minor injuries such as ankle sprain to the limbs. The pathophysiologic mechanism of CRPS is unknown [24]. Characteristically, there is interplay between peripheral and central pathophysiology. Limb signs (such as swelling/sweating and color/temperature changes) usually reduce with time, even when pain and motor symptoms persist [24,25]. However, such a reduction of limb signs is in itself not ‘recovery.’ Where pain persists, the condition is best considered to be active. There is no proven cure for CRPS. Approximately 15% of individuals will have unrelenting pain and physical impairment two years after the onset of CRPS, and the condition is considered chronic [26,27,28]. Nevertheless, accurate differential diagnosis of chronic post-traumatic CRPS is important because it can avoid unnecessary opioid misuse/addiction, secondary physical problems associated with the disuse of the affected limb, and psychological consequences; also, it can enhance pain management and provide appropriate rehabilitation training. 

Establishing the diagnosis of CRPS based solely on the clinical symptoms and radiologic finding is challenging. It is hard to differentiate chronic post-traumatic CRPS from other diseases based only on clinical criteria, and clinical evaluation is somewhat subjective with poor inter-observer reliability [29]. In particular, the positive and negative predictive values of TPBS were low to moderate in distinguishing between usual post-traumatic periarticular changes and CRPS [18]. SPECT (single photon emission computed tomography)/CT (computed tomography) is an imaging modality for evaluating extremity pain. SPECT/CT has an additional role in excluding other diagnoses such as arthritis, pseudoarthropathy, benign or malignant bony lesions, and even metabolic bone diseases such as Paget disease [30,31]. However, no studies are using SPECT/CT to evaluate the diagnostic performance of CRPS. Possible disadvantages of DITI include low sensitivity; the temperature can vary from one point to another within the same area, and temperatures are not constant over time [32]. Therefore, objective test results should be considered in the diagnosis. To the best of our knowledge, this is the first study to compare and analyze the diagnostic performance of TPBS and DITI targeting only on the chronic stage of CRPS that has been caused by trauma for more than one year. 

In this study, TPBS and DITI were compared in patients who complained of CRPS symptoms after an injury while in obligatory military service and had been tested more than one year after injury. There was a significant positive correlation between the count ratio on blood pool imaging of TPBS and color grade on the affected side of DITI (Spearman’s rho = 0.4810, 95% CI = 0.01826 to 0.7741, *p* = 0.0433). However, there was no significant positive or negative correlation between the count ratio in blood flow and delayed imaging of TPBS and the color grade of skin temperature in DITI. Our study identified that the TPBS showed significant results for diagnosing the chronic post-traumatic CRPS in both the qualitative analysis using uptake pattern and the quantitative analysis using the uptake ratio of the affected and unaffected sides. There was no significant difference in the color grade of the affected side on DITI between the CRPS and non-CRPS groups. Regarding SP or PLR, combined criteria 2 showed the highest impact, and were significantly different from other combined criteria. The specificity of combined criteria 2 was the highest at 92.3% and was significantly higher than that of combined criteria 1. In contrast, the sensitivity of combined criteria 1 was the highest at 94.4%, statistically significantly higher than that of combined criteria 2 and its NLR was the lowest. This study was aimed at patients who came to our hospital for a physical examination to be registered as veteran compensation recipients. Based on these diagnostic results, we can use the two tests appropriately, depending on the situation. In other words, when the purpose of screening is to find as many positive cases as possible using the sensitive combined criteria 1, it is advisable to do an additional workup. This will reduce the number of individuals that are deprived unfairly of compensation and treatment opportunities due to not being registered as veterans. On the other hand, when it is expected that various additional legal or social benefits will be obtained through the diagnosis of chronic post-traumatic CRPS, the high specificity of combined criteria 2 will help reduce false positives and social costs. It can be used for the objective evaluation of subjects by presenting imaging diagnostic criteria with high specificity and contribute to a reduction in unnecessary health costs. 

In this study, the TPBS pattern of 18 patients diagnosed with CRPS according to the Budapest criteria was D-D-D (10/18, 55.6%), S-S-S (3/18, 16.7%), S-S-I (2/18, 11.1%), I-I-I (2/18, 11.1%), and D-D-S (1/18, 5.6%). These results are consistent with secondary changes in neurovascular transmission that may cause vasoconstriction in the chronic stage [33]. In 6 out of 10 patients in D-D-D, the color grade of the affected side in DITI was grade 1 to 3. On the other hand, the remaining four patients in D-D-D had a color grade of grade 10 or higher and skin temperature was maintained. Vasoconstriction is associated with the development of chronic CRPS, but other mechanisms also contribute. There is a possibility that cold and warm CRPS coexist regardless of duration [10]. The scintigraphic pattern depends on the duration or stage of the disease [34]. The classic findings of increased flow and uptake in TPBS may be absent in several situations. Chronic cases of CRPS may also show variable and atypical patterns. As CRPS becomes chronic, there is a tendency for blood flow and uptake on delayed images to decrease, resulting in mixed and atypical patterns [35]. Among them, a clinically problematic case is when chronic post-traumatic CRPS shows an S-S-S pattern [i.e., symmetric uptake during all three phases] on TPBS and is difficult to distinguish from normal findings. In a study by Moon et al., 35.3% (6/17) of patients had an S-S-S pattern 12 months or longer after symptom onset. In our study, 15% (3/20) of the 20 patients (CRPS and non-CRPS group) had the S-S-S pattern, and all three cases had a low skin temperature of less than grade 8 on the affected side in DITI. If the criterion was that chronic post-traumatic CRPS occurred only when the color grade of skin temperature on the affected side was less than 8 in patients with the S-S-S pattern, it was possible to significantly differentiate between non-CRPS and CRPS groups, and SN 100%, SP 70.6%, AC 75%, PLR 3.4 (95% CI, 1.628 to 7.101), and NLR 0. Therefore, if the pattern shows S-S-S and the color grade of the body surface temperature on the affected side is more than 8, we can confidently say that there is no chronic post-traumatic CRPS. Even though the DITI was not statistically significant if S-S-S pattern is shown, to some extent a differential diagnosis for patients who are not chronic post-traumatic CRPS can be made confidently.

There were several limitations in our study. First, this is a retrospective single-center study and all subjects were male, which may induce selection bias. Secondly, the study population was a small size, particularly for patients with chronic post-traumatic CRPS, because it is a rare disease. Thirdly, we used the Budapest research criteria to discriminate between CRPS and non-CPRS, and these criteria have not been officially endorsed. Fourthly, our data were cross-sectional and were only gathered one time from each patient. Despite these limitations, our study remains one of the most extensive thermographic studies on patients with chronic post-traumatic CRPS. Therefore, further prospective studies with larger numbers of participants are warranted to investigate the diagnostic value of our suggested imaging criteria for the chronic post-traumatic CRPS. 

In summary, TPBS can be useful in diagnosing chronic post-traumatic CRPS. Moreover, we can suggest that different diagnostic criteria be applied depending on the purpose. When it is necessary to increase the sensitivity, chronic post-traumatic CRPS should be considered if either TBPS or DITI is abnormal. However, if there are legal problems related to post-traumatic CRPS, or if unnecessary economic or social benefits are expected from being diagnosed, the diagnostic criteria considering both the pattern analysis and quantitative analysis in TPBS should be considered.

## Figures and Tables

**Table 1:** Characteristics of enrolled subjects.

	Variables	CRPS Group (*n* = 18)	Non-CRPS Group (*n* = 26)
	Sex (Male: Female)	18:0	26:0
	Age (years)	26.7 + 3.4	26.8 + 3.5
	Disease duration (days)	1142.6 + 736.9	1163.7 + 747.9
	Weight (kg)	74.8 + 5.2	76.3 + 3.8
	Height (cm)	173.8 + 6.1	174.1 + 3.2
	Gender (Male, %)	100	100
	Ethnicity (Korean, %)	100	100
Symptoms	Sensory	94.4% (17/18)	53.8% (14/26)
	Vasomotor	77.8% (14/18)	76.9% (20/26)
	Sudomotor/Oedema	88.9% (16/18)	73.1% (19/26)
	Motor/Trophic	88.9% (16/18)	50.0% (13/26)
Signs	Sensory	94.4% (17/18)	23.1% (6/26)
	Vasomotor	61.1% (11/18)	42.3% (11/26)
	Sudomotor/Oedema	66.7% (12/18)	19.2% (5/26)
	Motor/Trophic	77.8% (14/18)	11.5% (3/26)

CRPS = Complex regional pain syndrome.

**Table 2:** Comparison of three-phase bone scintigraphy and digital infrared thermography imaging findings between CRPS and non-CRPS.

		CRPS Group	Non-CRPS Group	*p* Value
TPBS (Pattern analysis)	Positive	15	9	**0.002**
	Negative	3	17	
TPBS (Uptake ratio on pool phase ≤0.805)	Positive	13	3	**0.011**
	Negative	5	23	
DITI	Positive	11	12	0.334
	Negative	1	9	

CRPS = Complex regional pain syndrome, TPBS = Three-phase bone scintigraphy, DITI = Digital infrared thermography imaging. Statistically significant (*p* < 0.05) values are in bold.

**Table 3:** Uptake ratios of affected side to unaffected side in each phase of bone 3 phase and difference of color grade in DITI between CRPS and non-CRPS group.

	Findings	CRPS Group (*n* = 18)	Non-CRPS Group (*n* = 26)	*p* Value
Uptake ratio on TPBS	Flow phase	0.91 + 0.41	0.89 + 0.40	0.857
	Blood pool phase	0.75 + 0.34	0.98 + 0.23	0.011
	Delayed phase	0.97 + 0.29	0.96 + 0.29	0.100
	Grade difference in DITI	2.11 + 2.46	2.17 + 2.50	0.138

CRPS = Complex regional pain syndrome, TPBS = Three-phase bone scintigraphy, DITI = Digital infrared thermography imaging.

**Table 4:** Diagnostic performance of TPBS and DITI for diagnosing chronic post-traumatic CRPS.

	SN	SP	AC	PLR	NLR	*p* Value
TPBS (Pattern analysis)	83.3% (15/18)	65.4% (17/26)	72.7% (32/44)	2.407	0.255	**0.002**
TPBS (Uptake ratio on blood pool phase ≤0.805)	72.2% (13/18)	88.5% (23/26)	81.8% (36/44)	6.259	0.314	**0.011**
Combined criteria 1	94.4% (17/18)	56.3% (9/16)	59.1% (26/44)	1.444	0.160	**0.0020**
Combined criteria 2	61.1% (11/18)	92.3% (24/26)	79.5% (35/44)	7.944	0.421	**0.0002**

TPBS = Three-phase bone scintigraphy, DITI = Digital infrared thermography imaging, CRPS = Complex regional pain syndrome, Combined criteria 1 = uptake pattern of I-I-I, S-S-I, D-D-I, D-D-S, or D-D-D in TPBS or a difference in one or more color grades in DITI, Combined criteria 2 = uptake pattern of I-I-I, S-S-I, D-D-I, D-D-S, or D-D-D and ratio on blood pool phase ≤0.805 in TPBS. Statistically significant (*p* < 0.05) values are in bold.

## Data Availability

The data that support the findings of this study are available from the corresponding author M.C., upon reasonable request.
